# Supplementary material for: Interactions between earliest Linearbandkeramik farmers and central European hunter gatherers at the dawn of European Neolithization
Source: Sci Rep. 2019 Dec 20;9:19544. doi: 10.1038/s41598-019-56029-2 (PMC6925266; doi:10.1038/s41598-019-56029-2)
Supplement: Supplementary file 1 — Supplementary Information [file 41598_2019_56029_MOESM1_ESM.pdf]

Interactions between earliest *Linearbandkeramik* farmers and central European hunter gatherers at the dawn of European Neolithization

Alexey G. Nikitin, Peter Stadler, Nadezhda Kotova, Maria Teschler-Nicola, T. Douglas Price, Jessica Hoover, Douglas J. Kennett, Iosif Lazaridis, Nadin Rohland, Mark Lipson, and David Reich

Supplementary Information

Supplementary Figure S1.
A zoomed-in PCA plot from Figure 1, showing the position of Individuals 1 (I6912) and 3 (I6914) relative to Anatolian and European Neolithic farmers. ………………………………………………………… 2

Supplementary Table S1. Library-level metrics for Brunn Wolfholz site 2 genetic data. ………… 3

Supplementary Figure S1. A zoomed-in PCA plot from Figure 1, showing the position of Individuals 1 (I6912) and 3 (I6914) relative to Anatolian and European Neolithic farmers.

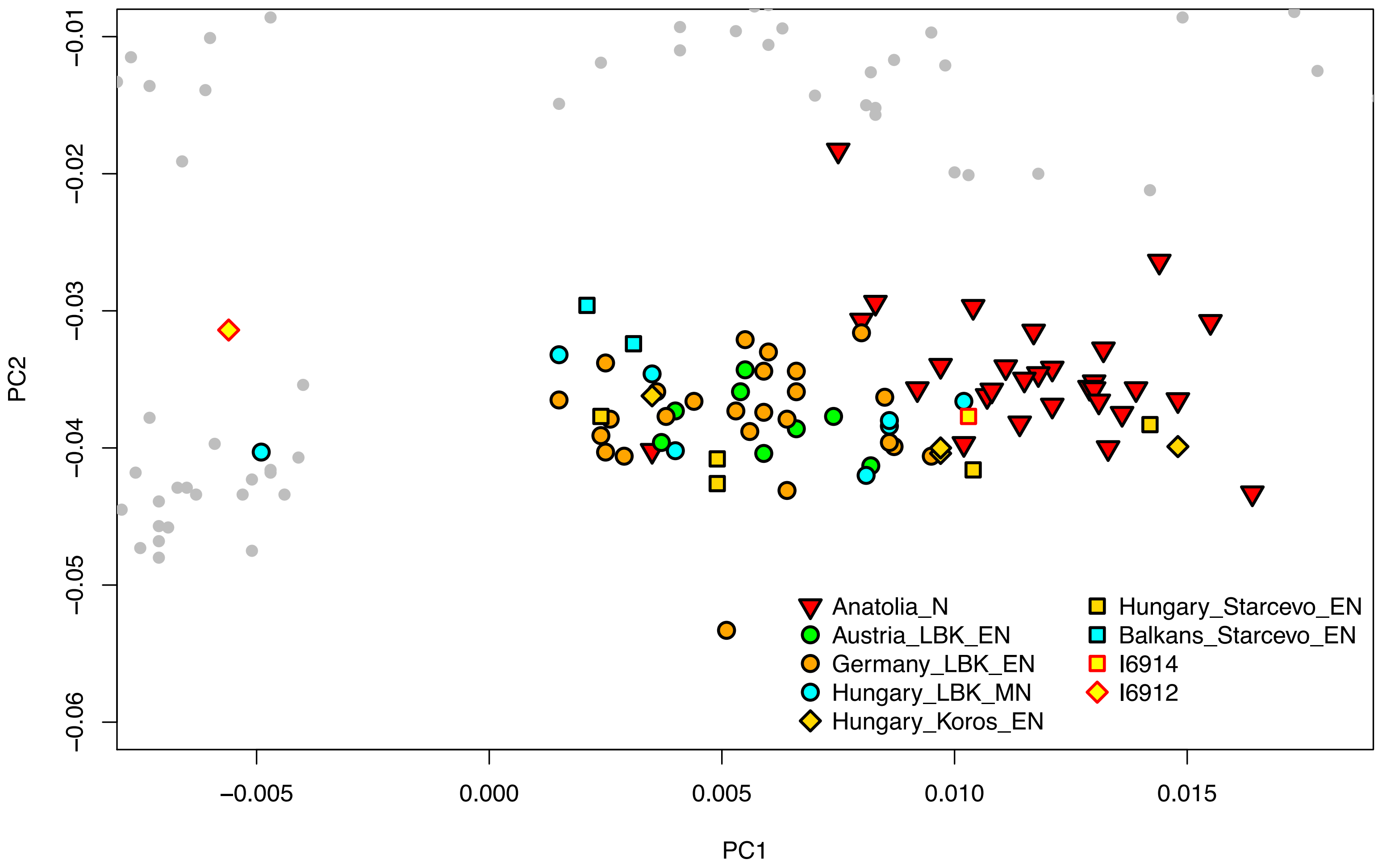

Supplementary Table S1. Library-level metrics for Brunn Wolfholz site 2 genetic data.

**Library ID**	S6912.E1.L3	S6912.E1.L2	S6912.E1.L3	S6912.E1.L5	S6912.E1.L6	S6913.E1.L1	S6914.E1.L1	S6915.E1.L1
**Lab Ind. ID**	I6912	I6912	I6912	I6912	I6912	I6913	I6914	I6915
**Skeletal Code/Element**	Ind. #1, left lower first or second molar (permanent tooth no. 36 or 37)					Ind. #2, left or right lower second molar (permanent mandibular molar no. 37 or 47)	Ind. #3, right lower second molar (permanent mandibular molar no. 47)	Ind. #4, left lower second molar (permanent mandibular molar no. 37)
**Powder Extracted (mg)**	69	69	69	69	69	73	82	76
**Library protocol**	Double-stranded	Double-stranded	Single-stranded	Double-stranded	Single-stranded	Double-stranded	Double-stranded	Double-stranded
**UDG Treatment**	partial	minus	minus	partial	partial	partial	partial	partial
**uL Extract Used**	10	10	10	10	10	10	10	10
**mg Equivalent Powder Used**	7.7	7.7	7.7	7.7	7.7	8.1	9.1	8.4
**mtDNA Raw Sequences**	1540475	4895353	5569976	4363797	5222170	1763868	1574760	1628891
**mtDNA Sequences Passing Filters**	1328578	4280940	4539284	3394382	4031310	1557034	1443480	1320302

**mtDNA Sequences Aligning**	344350	33525	61320	28964	61225	393193	46072	2623
**mtDNA Sequences Aligning PostDedup**	19275	17161	36981	13126	35607	58380	18405	346
**mtDNA Coverage**	54.4	55.5	127.3	39.7	111.8	187.0	48.7	0.9
**mtDNA Median [Mean] Seq Length**	45	[53.6]	[57.1]	[50.1]	[52.0]	50	43	43
**mtDNA Damage Last Base**	0.13	0.45	0.47	0.13	0.14	0.08	0.14	0.11
**mtDNA Consensus Match**	1.000	0.984	0.955	0.998	0.976	1.000	0.961	0.918
**mtDNA Consensus Match 95CI**	[0.997,1.000]	[0.963, 0.994]	[0.932, 0.973]	[0.990, 1.000]	[0.962, 0.986]	[0.998,1.000]	[0.951,0.968]	[0.838,0.967]
**mtDNA Haplogroup**	J1	J1	J1c8	J1	J1c	U5a1	K1b1a	..
**Nuclear Raw Reads**	4142974	4895353	5569976	4363797	5222170	4716548	11664837	5602849
**Nuclear Sequences Merged with Barcode**	3456700	4280940	4539284	3394382	4031310	4092020	10590010	4933624

**Nuclear Target Sequences Passing QC PreDedup**	76508	13533	26209	10776	32522	52727	2709080	10521
**Nuclear Target Sequences Passing QC PostDedup**	5222	4751	12941	3298	15246	7699	642592	1219
**Nuclear Unique SNPS Hit**	4830	3866	10888	3351	15488	7223	429000	..
**Nuclear Coverage at Targeted Positions**	0.004	0.004	0.011	0.003	0.013	0.006	0.497	..
**Nuclear Median [Mean] Seq Length**	43	[58.8]	[64.7]	[55.9]	[62.4]	39	44	37
**Nuclear Damage Last Base**	0.03	0.32	0.32	0.09	0.08	0.03	0.10	0.01
**Nuclear X Hits**	103	77	303	56	333	150	9551	..
**Nuclear Y Hits**	73	40	142	25	135	107	7109	..
**Nuclear Sex**	M	M	M	M	M	M	M	..
**Nuclear ANGSD SNPs**	1	0	0	1	4	0	345	0
**Nuclear ANGSD X-contam Mean**							0.0044	

**Nuclear ANGSD X-contam Z-score**	0.8999
